# Spatial distribution of *Plasmodium knowlesi* cases and their vectors in Johor, Malaysia: in light of human malaria elimination

**DOI:** 10.1186/s12936-021-03963-0

**Published:** 2021-10-29

**Authors:** Sandthya Pramasivan, Romano Ngui, Nantha Kumar Jeyaprakasam, Jonathan Wee Kent Liew, Van Lun Low, Norzihan Mohamed Hassan, Wan Yusoff Wan Sulaiman, Ropiah Jaraee, Roslinda Abdul Rahman, Jenarun Jelip, Indra Vythilingam

**Affiliations:** 1 Department of Parasitology, Faculty of Medicine, Universiti Malaya, Kuala Lumpur, Malaysia; 2 Malaria Research Centre, Faculty of Medicine and Health Sciences, Universiti Malaysia Sarawak (UNIMAS), 94300 Kota Samarahan, Sarawak, Malaysia; 3 Tropical Infectious Diseases Research & Education Centre (TIDREC), Universiti Malaya, Kuala Lumpur, Malaysia; 4 Entomology & Pest Sector, Disease Control Division, Ministry of Health Malaysia, Putrajaya, Malaysia; 5 Entomology & Pest Unit, Johor State Health District Department, Johor, Malaysia; 6 Kota Tinggi District Health Office, Johor, Malaysia; 7 Disease Control Division, Ministry of Health Malaysia, Putrajaya, Malaysia; 8 Present Address: Environmental Health Institute, National Environment Agency, Singapore, Singapore

**Keywords:** Knowlesi malaria, *Anopheles*, GIS, *COI*, *ITS2*, Spatial distribution, Johor, Malaysia, *Plasmodium knowlesi*, Leucosphyrus

## Abstract

**Background:**

*Plasmodium knowlesi,* a simian malaria parasite infection, increases as *Plasmodium falciparum* and *Plasmodium vivax* infections decrease in Johor, Malaysia. Therefore, this study aimed to identify the distribution of vectors involved in knowlesi malaria transmission in Johor. This finding is vital in estimating hotspot areas for targeted control strategies.

**Methods:**

*Anopheles* mosquitoes were collected from the location where *P. knowlesi* cases were reported. Cases of knowlesi malaria from 2011 to 2019 in Johor were analyzed. Internal transcribed spacers 2 (*ITS2*) and cytochrome c oxidase subunit I (*COI*) genes were used to identify the Leucosphyrus Group of *Anopheles* mosquitoes. In addition, spatial analysis was carried out on the knowlesi cases and vectors in Johor.

**Results:**

One hundred and eighty-nine cases of *P. knowlesi* were reported in Johor over 10 years. Young adults between the ages of 20–39 years comprised 65% of the cases. Most infected individuals were involved in agriculture and army-related occupations (22% and 32%, respectively). Four hundred and eighteen Leucosphyrus Group *Anopheles* mosquitoes were captured during the study. *Anopheles introlatus* was the predominant species, followed by *Anopheles latens*. Spatial analysis by Kriging interpolation found that hotspot regions of *P. knowlesi* overlapped or were close to the areas where *An. introlatus* and *An. latens* were found. A significantly high number of vectors and *P. knowlesi* cases were found near the road within 0–5 km.

**Conclusions:**

This study describes the distribution of *P. knowlesi* cases and *Anopheles* species in malaria-endemic transmission areas in Johor. Geospatial analysis is a valuable tool for studying the relationship between vectors and *P. knowlesi* cases. This study further supports that the Leucosphyrus Group of mosquitoes might be involved in transmitting knowlesi malaria cases in Johor. These findings may provide initial evidence to prioritize diseases and vector surveillance.

**Supplementary Information:**

The online version contains supplementary material available at 10.1186/s12936-021-03963-0.

## Background

Malaria cases have declined over the years but still pose a significant public health concern in many tropical countries. Two hundred and twenty-nine million malaria cases were reported worldwide in 2019, as reported by World Health Organization (WHO). Globally, malaria case incidence decreased by 27% between 2000 and 2015 and by less than 2% between 2015 and 2019, reflecting a slowdown of the rate of decline since 2015 [1]. Around 3% of the global burden of malaria cases were in the WHO South-East Asia region. Malaria cases have declined by 73%, from 23 million in 2000 to about 6.3 million in 2019 [1]. In 2019, the WHO Western Pacific Region had an estimated 1.7 million cases, declining 43% from 3 million cases in 2000. Malaria case incidence decreased from five to two cases per 1000 people at risk over the same period [1]. In Southeast Asia, malaria has decreased due to intensive malaria control activities and increased political commitment, and these countries have targeted malaria elimination for 2030 [2]. Malaysia has eliminated human malaria cases by 2020 [3, 4] and is awaiting elimination status from the WHO. However, *Plasmodium knowlesi,* a simian malaria parasite is currently the predominant species affecting humans in Malaysia [5–11]. About one-third (32%) of the total knowlesi cases occur in Peninsular Malaysia, and most of these occur in central, south-eastern, and northern coastal regions [7].

*Plasmodium knowlesi* is transmitted by *Anopheles* mosquitoes between non-human primate hosts and humans. Approximately 70 species of *Anopheles* can transmit malaria parasites in nature, and 41 are considered dominant vector species, of which 19 are found in Asia [12, 13]. The principal vectors of *P. knowlesi in* Malaysia are *Anopheles introlatus* in Selangor*, Anopheles cracens* in Pahang, *Anopheles latens* in Sarawak, and *Anopheles balabacensis* in Sabah [9, 14–16].

The first natural infection of *P. knowlesi* was reported from Pahang, Malaysia in 1965 [17], and the second was from Johor, Malaysia in 1971 [18]. The state of Johor consists of ten districts, and for the past 9 years, the number of *P. knowlesi* cases in Johor were high, especially in Kluang, Mersing, and Kota Tinggi districts (Records from State Health Department). However, in these three districts, the vectors involved in the transmission of knowlesi malaria and their distribution remain unknown. Hence, there is a daunting array of challenges and unknowns.

Thus, this study aimed to highlight the spatial distribution of the knowlesi malaria cases and their vectors throughout the state of Johor. With this information, it may be feasible to perhaps design control measures that can be instituted to control simian malaria in the future.

## Methods

### Study sites

**Fig. 1:**
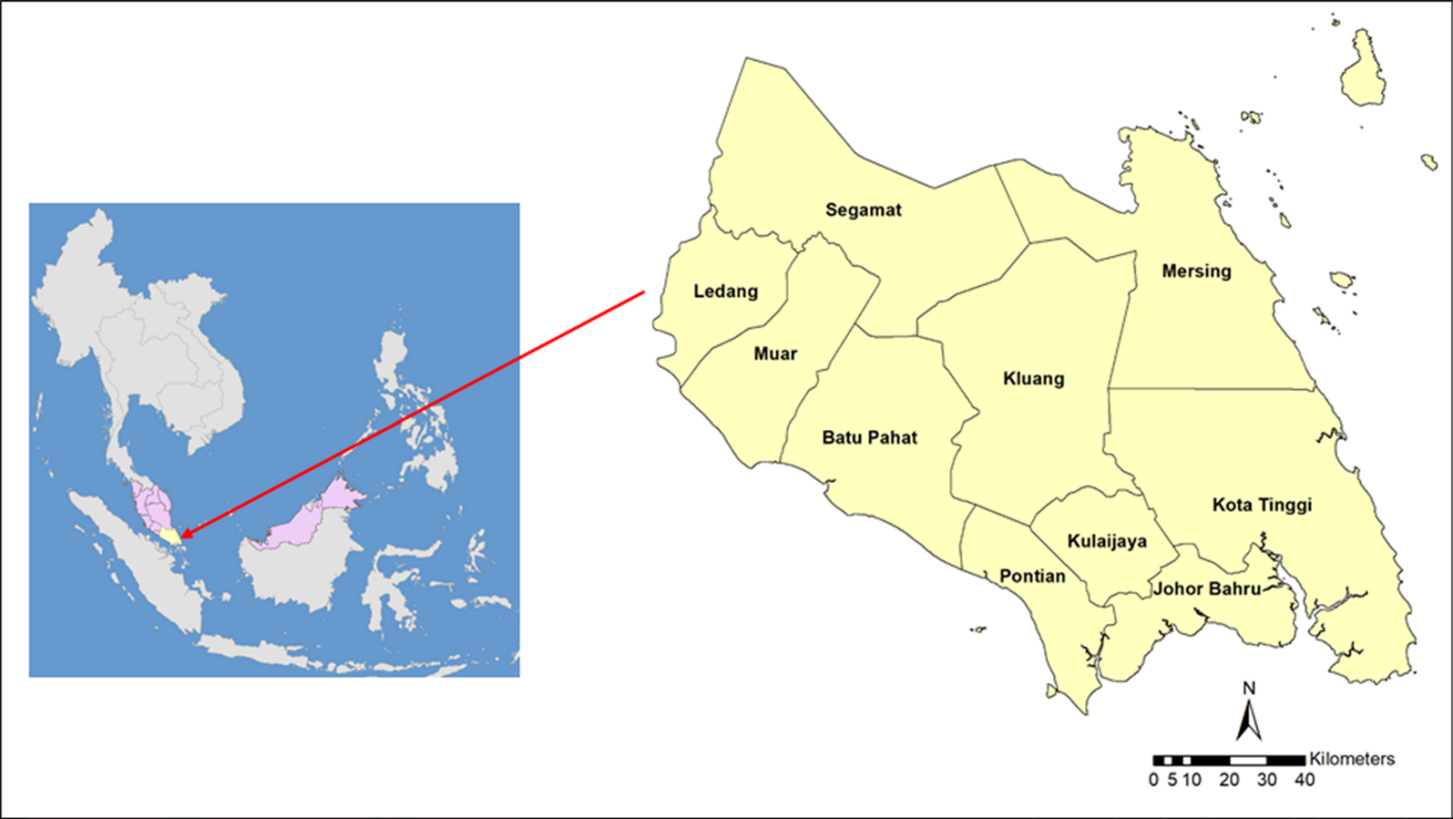
Map showing Malaysia and the state of Johor within Southeast Asia

This study was conducted in the state of Johor, located in the south of Peninsular Malaysia. It is linked to Singapore by a causeway. It is known for its beaches, national parks, mountainous jungles, and wildlife. Johor has an equatorial climate and a highly diverse rainforest. This state consists of ten districts (Fig. 1). Since most cases were reported in Kluang, Kota Tinggi, and Mersing, these districts were the targets for vector studies. The majority of these three districts are covered with dense forest which is classified as natural inland forest, peat swamp forest, mangrove, and Nipah forest. The local people are primarily involved in agricultural activities, fishing, factories, and small businesses. There are also tribal peoples (Orang Asli) living in these districts.

### Data of malaria cases in Johor Sate from 2011–2019

Records of all malaria cases from 2011 to 2019 in the state of Johor were provided by Johor State Health Office. According to their records all knowlesi malaria cases, were confirmed by the Institute for Medical Research (IMR), the Ministry of Health, Malaysia (MOH), by Polymerase Chain Reaction (PCR). In addition, the information regarding the age, gender, race/nationality, profession, address, home address, source of infection was gathered while performing case investigation. The latitude and longitudinal coordinates of the source of infection was compiled and included in the database.

### Mosquito collection, identification, and dissection

Mosquito collections were conducted in Kluang, Kota Tinggi, and Mersing for 17 nights, in June, September, and November of 2019, March and July of 2020. Various places were surveyed based on reports of the knowlesi malaria cases. The collections sites were chosen based on possible areas where people could have been infected and the distribution of macaques. All collection sites were located in the forest fringe, private plantation area, and rural villages where fishing and agricultural activities occurred. All the Leucosphyrus Group mosquitoes caught during this study were used for the analysis and interpolation from the forested area, which were accessible by roads.

Mosquitoes were collected using bare-leg capture (BLC) [19] as well as human baited trap, CDC light trap, and mosquito magnet from 1800 to 2330 as described in [20]. *Anopheles* were identified using the keys of Reid [21] and Sallum [22].

### DNA extraction and PCR

All *Anopheles* from the Leucosphyrus Group were further molecularly characterized. According to the manufacturer's protocol, DNA was extracted from the mosquitoes' legs by using InstaGene Matrix (Bio-Rad, California, USA). The extracted DNA was kept at − 20 °C until required. PCR targeting the internal transcribed spacer 2 (*ITS2*) and mitochondrial cytochrome c oxidase subunit I (*COI*) genes were carried out. The *ITS2* gene was amplified by ITS2A and ITS2B primers [23]. The PCR conditions were as follows: denaturation at 95 °C for 2 min, 35 cycles of amplification at 95 °C for 30 s, annealing step at 51 °C for 30 s with elongation step at 72 °C for 1 min, followed by final elongation step of 10 min at 72 °C. For amplification of the *COI* gene, the primers used were LCO1490 and HCO2198 primers [24]. The PCR conditions were as follows: denaturation at 95 °C for 3 min, 35 cycles of amplification at 95 °C for 1 min, annealing step at 50 °C for 1 min with elongation step at 72 °C for 1 min, followed by final elongation step of 10 min at 72 °C and a held at a temperature of 4 °C. Each reaction mixture of 25 μL contained 5 μL DNA template, 0.5 μM primers, respectively, 0.2 mM dNTP, 3 mM MgCl_2_, 1 × GoTaq® Flexi Buffer, and 1.0 U of GoTaq® DNA polymerase (Promega Corporation, Madison, WI, USA). This reaction mixture was applied to both primer sets. Amplicons were subjected to electrophoresis on 1.5% agarose gels. The amplified product was purified from the gel and sequenced.

### Sequence analysis

The *ITS2* and *COI* gene sequences from representative *An. introlatus* and *An. latens* samples collected from separate areas in this study were used. Sequences were aligned with other deposited sequences obtained from the NCBI GenBank using BioEdit (Version 7.2). A phylogenetic tree was generated using maximum-likelihood (ML) using the MEGA- X (Version 10.1.8) software with 1000 bootstrap replicates. Sequences were deposited in the NCBI GenBank (*ITS2* region of *An. latens*, MW587948-MW587956; *ITS2* region of *An. introlatus*, MW587822-MW587832; *COI* region of *An. latens*, MW585357- MW585364; *COI* region of *An. introlatus,* MW585345- MW585356).

### Spatial distribution of *Anopheles* species and *P. knowlesi* cases

The *P. knowlesi* human cases from 2011 to 2019 were provided by the District Health Offices. The geographic coordinates of *P. knowlesi* cases were determined from the possible location of infection or the home address using a combination of various electronic resources, including Google Earth (http://www.google.com), GeoNet Names Server (http://earth-info.nga.mil) and Tageo (http://www.tageo.com). These sources are accessible online and provide varying degrees of coverage, functionality, and ease of use. Each of the identified locations from one source was consequently cross-checked against other sources to ensure consistency of the identified coordinates. All the digital data coordinate system was synchronized using World Geodetic System (WGS 1984), which serve as the x (longitude or east–west) and y (latitude or north–south) that allows geographic positions to be expressed anywhere around the world.

Successfully 95% of cases were located then plotted as point features, creating a new geographic information system (GIS) layer representing point locations of *P. knowlesi* cases in Johor. In cases that were unable to locate usually the information was too general about the place. The *P. knowlesi* cases data were mapped at the district and sub-district levels. The district and sub-district levels in which each case has been recorded were linked to the Johor boundary map referred to as the base map. This Johor boundary map was obtained from the Department of Surveying and Mapping, Malaysia [25]. The geo-positioned cases were then exported and stored into ArcGIS 10.3.1 software (Earth Science Resource Institute, Redlands, California, USA) [26] for further exploration and analysis.

### Road and river network analysis

A set of spatial data, including road and water bodies, was downloaded from DIVA-GIS, a free program providing spatial data for mapping and geographic data analysis [27]. The geo-referenced *P. knowlesi* cases, road, and water bodies spatial data were converted to RSO Kertau, expressed in meters from World Geodetic System (WGS 1984) to support uniform analysis. The layer of road and water bodies and the layer identifying the point locations of confirmed *P. knowlesi* cases were then used in tangent with the Nearing tool in ArcGIS to identify the road and water bodies nearest to each case. The Near tool determines the Euclidean or straight-line distance between features in one layer and the nearest feature in another. Its use emphasizes that mosquito is not usually obstructed during flight by natural or artificial features. Subsequently, the road and water bodies layer were used to create several other GIS layers based on the attached road class information. Using the Near tool, the distance from each *P. knowlesi* case to the nearest road and water body categories was calculated. A threshold of 6 km was used as a cut-off point as distances beyond this were far too dispersed to warrant further investigation [28]. Therefore, all distances calculated for analysis were expressed in kilometers (km). Data were analysed using ArcGIS version 10.3.1 and exported to Microsoft Excel 2007. Further data analysis was carried out using SPSS version 21 (Statistical Package for the Social Sciences) program for Windows (SPSS, Chicago, IL, USA). The level of statistical significance was set at P < 0.05.

### Spatial interpolation

The spatial interpolation tool was used to predict values using data from the number of *P. knowlesi* cases incorporated into the area map. This study used the Kriging interpolation method to examine trends and patterns from human malaria cases data. The kriging interpolation method is based on spatial statistics and the weighting of each point in matrix form. Human malaria cases data were classified by using colour ranges to indicate the level of the cases from very low (blue), low (green), moderate (yellow), high (orange), and very high (red). To potentially improve the visualization, environmental factors such as forest cover and forest loss data from the University of Maryland, Department of Geographical science from 2001 to 2019 [29] and elevation data from Earth explorer were included [30]. This data set, a collaboration between the GLAD (Global Land Analysis & Discovery) lab at the University of Maryland, Google, USGS, and NASA, measure areas of tree cover loss across all global land at approximately 30 × 30 m resolution.

## Results

### Malaria cases in Johor from 2011–2019

**Fig. 2:**
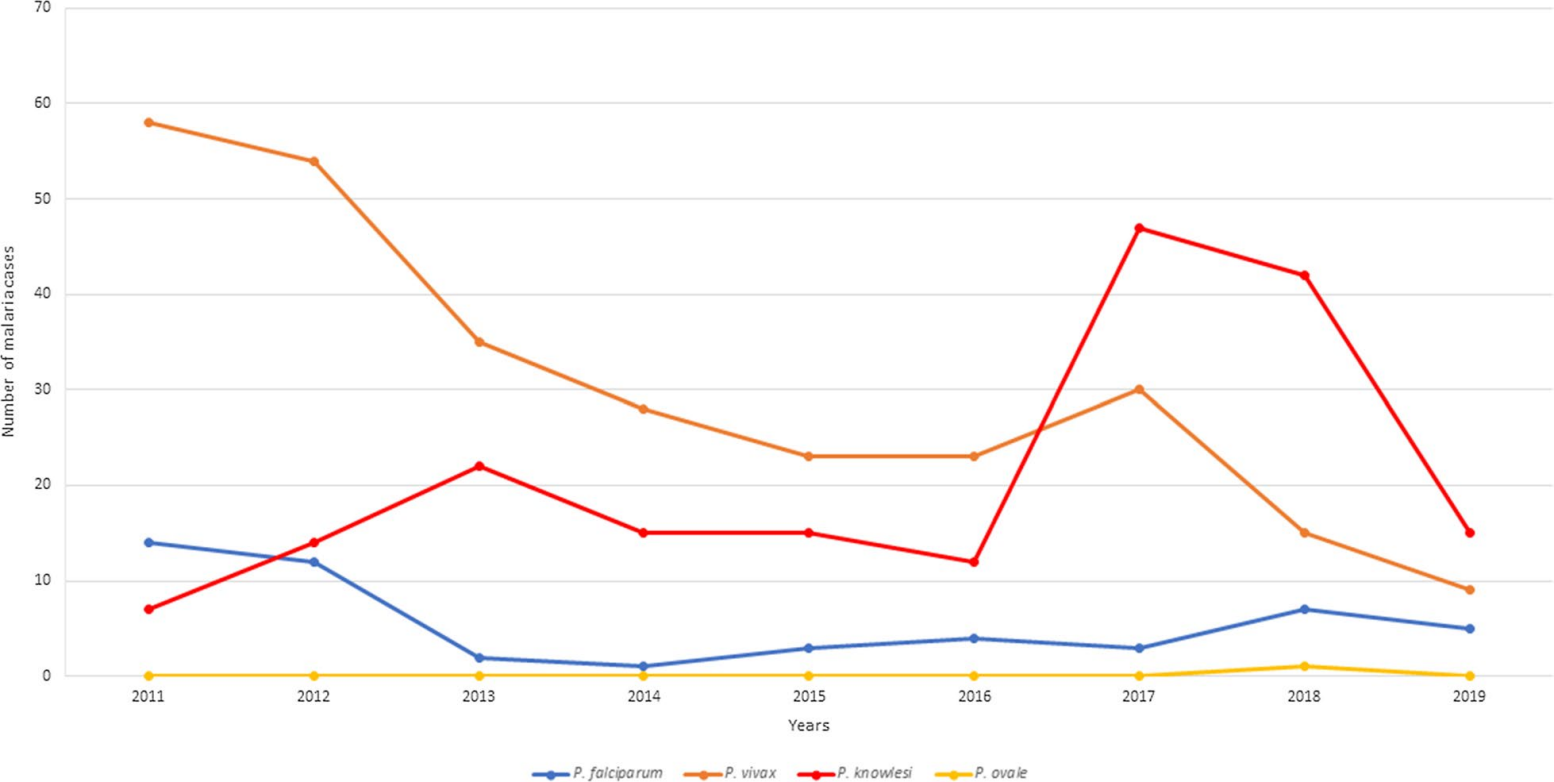
Malaria cases in Johor from year 2011–2019

A total of 516 cases of malaria were reported between 2011 and 2019 in the state of Johor, Malaysia. Of these, 275 (53.3%) were caused by *Plasmodium vivax*, 189 (36.6%) by *P. knowlesi*, 51 (9.9%) by *Plasmodium falciparum*, and 1 (0.2%) by *Plasmodium ovale*. *Plasmodium vivax* consistently contributed to the high number of cases, except for a decline in 2018–2019. However, from 2017, *P. knowlesi* cases increased substantially compared to the years prior and were the predominant species affecting humans (Fig. 2).

### Plasmodium knowlesi cases in Johore from 2011–2019

There were 189 knowlesi malaria cases over the past 9 years (i.e., 2011–2019). Of these, 179 (94.7%) cases involved males versus 10 (5.3%) females. Cases of *P. knowlesi* were predominant among the young adults (ages 20–39 years; 123 cases; 65.1%), followed by those above 40 years (35 cases; 18.5%). Most of the infected people were involved in the army and occupations related to agriculture (31.7; 21.2%), followed by people working in forested areas (8.5%) (Table 1).

**Table 1:** Characteristics of *P. knowlesi* cases reported in Johor State from year 2011–2019

Variables	2011	2012	2013	2014	2015	2016	2017	2018	2019	Total
Gender										
Male	6	14	20	14	15	12	43	41	14	179
Female	1	0	2	1	0	0	4	1	1	10
Age										
< 1–9	0	0	0	0	0	0	0	0	1	1
10–19	0	0	0	2	0	0	0	2	0	4
20–29	1	2	5	5	6	2	11	19	5	56
30–39	2	6	5	4	1	7	23	14	5	67
40–49	1	1	5	2	6	3	9	6	2	35
> 50	3	5	7	2	2	0	4	1	2	26
Occupation										
Agriculture	3	3	8	4	4	2	5	7	5	41
Construction	0	0	0	2	0	0	2	1	0	5
Forestry	0	1	2	1	2	1	4	3	2	16
Police/Army	0	4	2	3	1	6	15	23	6	60
Tourist	0	0	0	0	0	0	0	0	0	0
Mining	0	1	0	0	0	0	1	0	0	2
Explorer	0	1	1	0	0	0	0	0	0	2
Others	4	4	9	5	8	3	20	8	2	63

### Species composition of *Anopheles* mosquitoes

**Fig. 3:**
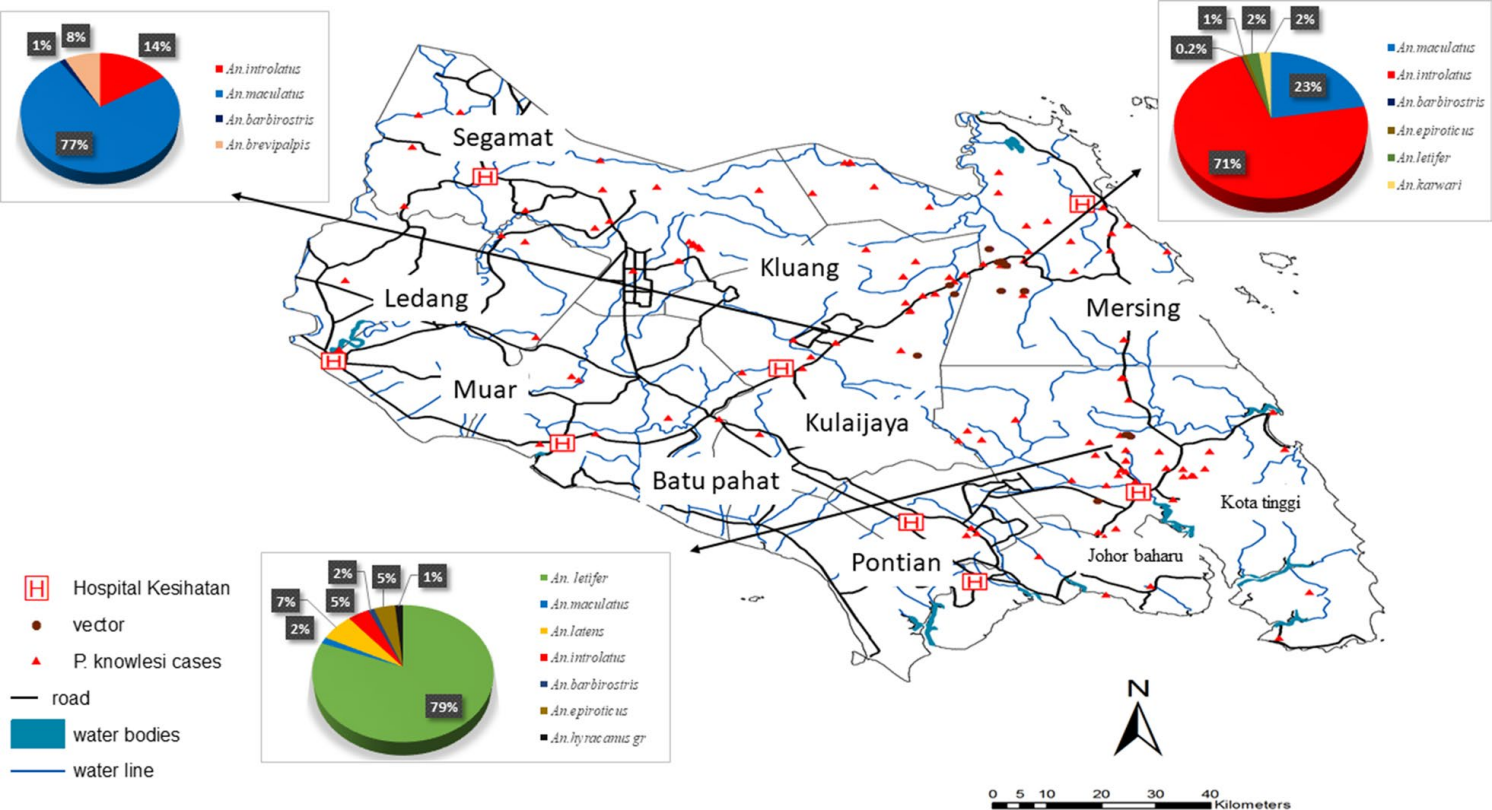
Spatial distribution of *P.knowlesi* cases and *Anopheles* species collected from Kluang, Kota Tinggi and Mersing districts between year 2019 to 2020

During the study, eight hundred and fifty-four mosquitoes belonging to nine species of *Anopheles* were collected, as shown in Fig. 3 and Additional File 1 Table S1. *Anopheles introlatus* was the predominant species (47%), followed by *Anopheles letifer* (25%) and *Anopheles maculatus* (15%). However, *An. letifer* was collected only in a particular site in vast quantities. *Anopheles latens* and *An. introlatus* were the only *Anopheles* belonging to the Leucosphyrus Group.

**Fig. 4:**
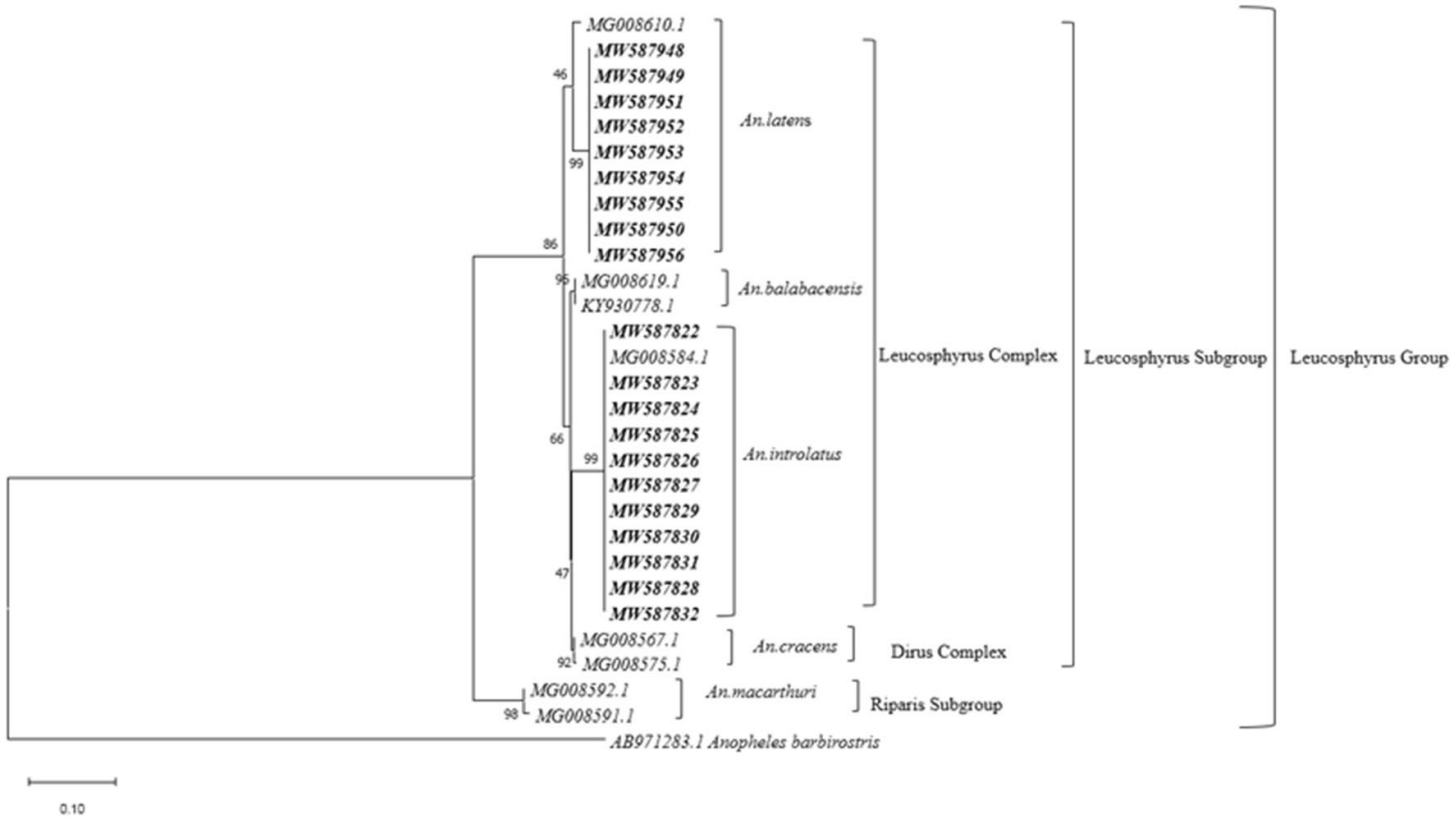
Maximum Likelihood analysis of Leucosphyrus group of *Anopheles* mosquitoes based on *ITS2* gene

**Fig. 5:**
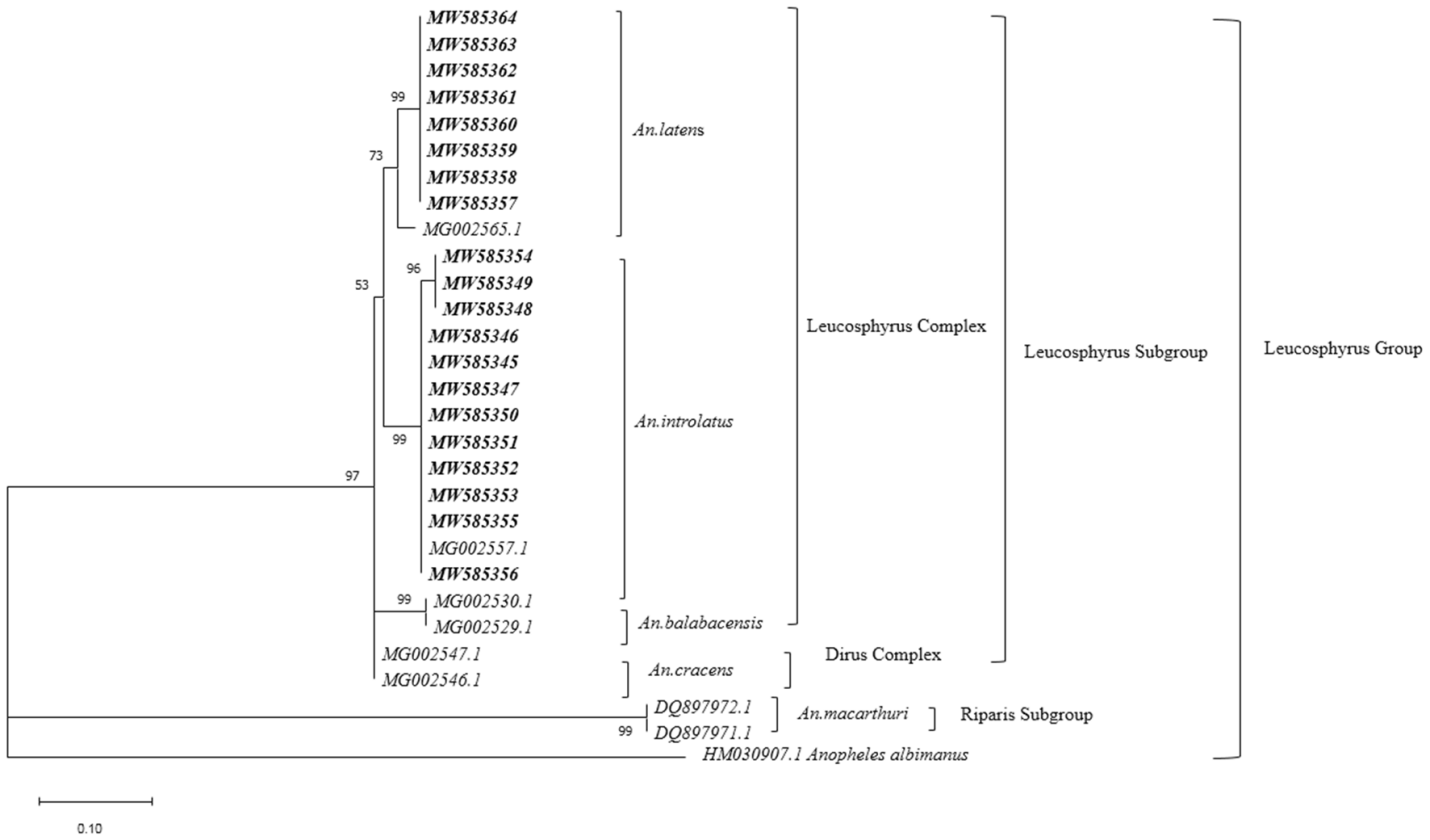
Maximum Likelihood analysis of Leucosphyrus group of *Anopheles* mosquitoes based on *COI* gene

Phylogenetic tree based on the ML approach showed that the *An. introlatus* collected in separate areas where known malaria cases occurred formed a monophyletic clade supported with substantial bootstrap value (Figs. 4 and 5). However, two clades of *An. latens* associated with East and West Malaysia populations were observed in both trees constructed from the *ITS2* and *COI* genes. Therefore, a further taxonomic study is warranted to clarify the species status of both clades.

### Distribution of the *Anopheles* species and *P. knowlesi* cases

The spatial distribution of the *P. knowlesi* cases and vectors collected near the water bodies and road in Johor is shown in Tables 2 and 3. Only *An. introlatus* and *An. latens* were included in the map (Fig. 3). Large numbers of *P. knowlesi* cases and vectors were found within 5 km distance radius from the water bodies and road.

**Table 2:** Number of *P. knowlesi* cases and their proximity to road and water bodies

Variables	Distance (km)						
Road	< 1	1.0–2.0	2.1–3.0	3.1–4.0	4.1–5.0	5.1–6.0	≥ 6.1
Primary^1^	45	12	4	6	2	1	6
Secondary^2^	40	26	9	12	3	4	10
Water							
Perennial^3^	51	52	25	14	9	19	13

^1^Primary road is a well-maintained road in a recognized system of highways

^2^Secondary road is an alternative way from the primary road, usually a two-way road for moderate or slow speed drive

^3^Perennial water body is a permanent water body that keeps full or flowing throughout the seasons

**Table 3:** Number of vectors and their proximity to road and water bodies

Variables	Distance (km)						
Road	< 1	1.0–2.0	2.1–3.0	3.1–4.0	4.1–5.0	5.1–6.0	≥ 6.1
Primary^1^	0	0	0	0	18	7	9
Secondary^2^	121	44	1	55	143	0	12
Water							
Perennial^3^	1	27	198	3	0	171	10

^1^Primary road is a well-maintained road in a recognized system of highways

^2^Secondary road is an alternative way from the primary road, usually a two-way road for moderate or slow speed drive

^3^Perennial water body is a permanent water body such as the sea that keeps full or flowing throughout the seasons

Tables 2 and 3 show the numbers of *P. knowlesi* cases and vectors and their proximity to water bodies and roads. The data in Table 2 demonstrate that many *P. knowlesi* cases occurred within 1 km from the road. The overall mean distance shows that there was a significant difference between the mean of *P. knowlesi* cases nearby to secondary/minor road (1.7 ± 1.5 km) than primary road/highways (1.1 ± 1.4 km) as assessed by the Mann–Whitney test (P < 0.05). The relationship between the road category and the *P. knowlesi* cases' frequency was further investigated using the Chi-square test of goodness-of-fit with a threshold of 6 km as a cut-off point. The result shows that a significantly higher number of *P. knowlesi* cases were found within 0–1 km away from the road [χ^2^ = 173.024, df = 5, (P < 0.05)]. The data in Table 3 shows a higher number of vectors were collected 5 km radius from the road. The mean distance shows that there was a significant difference between means of vector nearby to primary road/highways (6.3 ± 2.3 km) than secondary/minor road (2.9 ± 1.9 km) as analyzed by the Mann–Whitney test (P < 0.05). Chi-square test of goodness-of-fit was performed to investigate the relationship between road category and frequency of the number of vectors with a threshold of 6 km as a cut-off point. The result shows that significantly more vectors were found within 4.1–5.0 km away from the road [χ^2^ = 313.925, df = 5, (P < 0.05)].

The distribution of *P. knowlesi* cases was also analysed based on their distance to water bodies using the Kruskal–Wallis test. The results were not significant (df = 4, P > 0.05). However, further analysis to investigate the relationship between the number of vectors and the distance to the water body showed a significantly higher number of vectors collected within a 2.1–3.0 km radius (df = 5, P < 0.05).

**Fig. 6:**
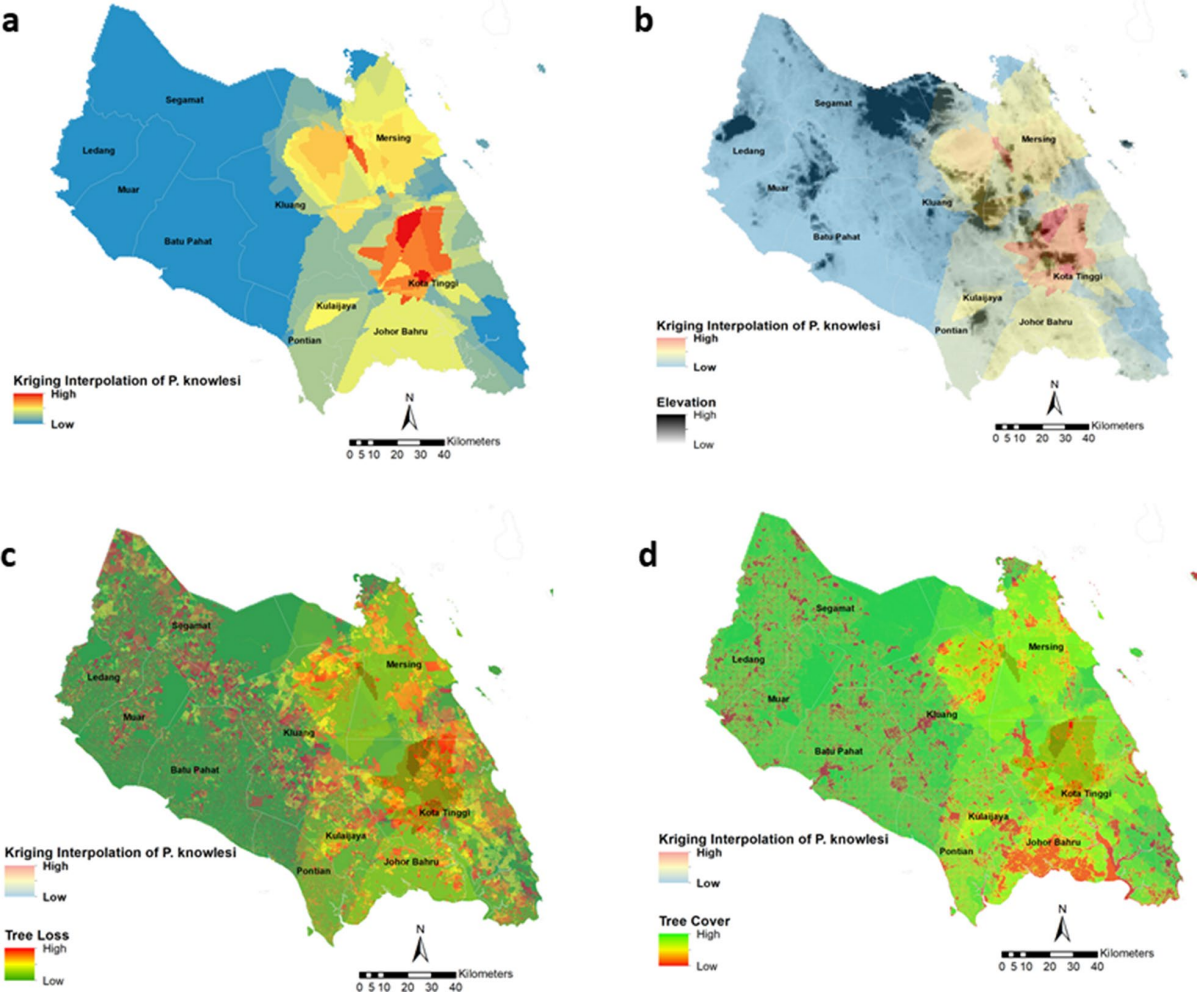
**a** Interpolated distribution of *P. knowlesi* cases in Johor using kriging interpolation method illustrating zonation of high and low area. **b**–**d** *P. knowlesi* cases overlaid with environmental factors. **b** *P. knowlesi* casesoverlaid with elevation; from high elevation (black) area to low elevation area (grey). **c** *P. knowlesi cases* overlaid with tree cover and; **d** tree loss from year 2011–2019

Figure 6a shows the *P. knowlesi* cases with kriging interpolation based on cases from 2011 to 2019. West part of Mersing and Northwestern Kota Tinggi had a high knowlesi malaria case (red). In contrast, the north part of Johor, such as Segamat, Tangkak, Muar, Batu Pahat, Northeastern Kota Tinggi, and South-eastern Mersing had the lowest malaria case (blue). The yellow zone areas (Johor Baharu and Mersing), orange zone areas (Kluang, Mersing, and Kulai), and red zone areas (Kota Tinggi and Mersing) were associated with highlands (Fig. 6b). Huge tree loss from 2009 to 2019 can be noticed all over the *P. knowlesi* case locations but mainly in the red zone areas (Fig. 6c and d).

## Discussion

From 2011 to 2019, 36.6% (189) of all malaria cases reported in Johor were caused by *P. knowlesi*. As the human malaria cases reduced over the years, the cases of *P. knowlesi* malaria have increased in Peninsular Malaysia [31]. Thus, the distribution and bionomics of simian malaria vectors are significant aspects required for zoonotic malaria prevention. While treating symptomatic patients is part of the malaria control programmes, vector control is important for malaria preventive measures. As the country moves towards malaria elimination, the surveillance system needs to include new tools to combat zoonotic malaria. Earlier studies in Peninsular Malaysia [14] and the current study demonstrate that different vectors are involved in this zoonotic transmission than human malaria. Thus, it is necessary to identify the vectors in order to be able to implement practical control steps. This study provides updated information and visualization on the distribution of *Anopheles* species and the knowlesi malaria cases in malaria-endemic regions in Johor.

Two *Anopheles* mosquito species from the Leucosphyrus Group: *An. introlatus* and *An. latens were obtained*. The predominant mosquito collected in this study was *An. introlatus,* and it is also a known simian malaria vector in Selangor, Peninsular Malaysia [14]. These mosquitoes were mainly collected in the three districts, despite the geographical variations between all the locations. *Anopheles introlatus* is distributed in West Malaysia, Indonesia, and Thailand [21, 22, 32].

On the other hand, *An. latens*, which was incriminated as a vector in Sarawak, Malaysia [15], was only found in minimal numbers in Kota Tinggi district. *Anopheles latens* is a forest breeding mosquito found in dense jungles and forest fringe [21, 33]. Mersing yielded a more significant number of Leucosphyrus Group of mosquitoes compared to both Kluang and Kota Tinggi districts. These high numbers captured in Mersing could be due to relative humidity, high altitude, tree cover, and shaded environment [34].

In the present study, *An. introlatus* and *An. latens* were infected with *Plasmodium inui* and *Plasmodium cynomolgi* (unpublished data). Based on previous studies [14, 15] it is likely that *An. introlatus* and *An. latens* are the vectors for *P. knowlesi* in the areas. In addition, kriging interpolation analysis showed that the hotspot areas of *P. knowlesi* cases overlapped with where these vectors were caught. This supports the assumption that *An. introlatus* and *An. latens* could be the vectors for the cases of *P. knowlesi* in the districts of Johor.

There is a positive correlation between the number of vectors collected and the road networks. High numbers of vectors were caught 5 km away from the road areas. More vectors were found within 3 km from water bodies. This may be due to the presence of temporary water puddles or water pockets formed within 2.1–3.0 km. The Leucosphyrus Group of *Anopheles* is forest dwelling mosquitoes commonly found in forested and agricultural areas [14].

Moreover, *Anopheles* larval habitats are in deeply shaded, clean, and natural water pockets or puddles near rivers [35]. Thus, higher numbers were obtained near forest or rural areas compared to places with road access. This study shows that the geostatistical kriging method can be used to estimate the spatial risk of malaria distribution in settings where spatial data are available. It shows spatial heterogeneity in the risk of malaria spread in Johor, suggesting that the infection did not affect all populations in the area at the same magnitude. Evidence has shown that variations in underlying socioeconomic, climatic, geographic factors and human activities could lead to spatial heterogeneity in the risk of malaria distribution [36–38]. Elevation, land cover, and area type were the most critical environmental/climatic variables responsible for malaria transmission [39]. Areas with forest cover had a more significant number of cases compared to more minor dense areas. This is because *Anopheles* is a forest-dwelling mosquito, which lives in a humid, shady, and moist environment [40]. This kind of landscape provides clean and suitable water bodies for the breeding of the *Anopheles* mosquitoes. Based on previous studies [31, 41, 42], the current results concur that people working in the jungle like police or army and people involved in the agricultural sector were more likely to be exposed to the infection than other occupations. The number of males infected with knowlesi malaria was higher than the female because of their type of profession. They were primarily farmers, loggers, forestry workers, and agricultural workers, at higher risk of exposure to *Anopheles* mosquitoes.

The long tail macaques (*Macaca fascicularis*), the natural host of *P. knowlesi* [43] and native to Peninsular Malaysia, have migrated to the forest fringes with deforestation, and possibly these mosquitoes may have joined the macaques and colonized forest fringes. The *Anopheles* Leucosphyrus Group of mosquitoes has been found as the primary vector of *P. knowlesi* with relatively high biting rates in farms, plantations, and forest fringe [9, 44, 45]. *Plasmodium knowlesi* malaria in Johor was reported mainly in the districts or areas where the clearing of the forest had taken place. In 1997, a similar pattern of malaria transmission attributed to deforestation was noticed in Perak, Peninsular Malaysia [46]. Since *Anopheles* mosquitoes are localized in the areas undergoing deforestation, people involved in that activity are highly exposed to the infections. Moyes [47] suggested that conversion of intact forest to disturbed forest could be a factor for zoonotic malaria transmission due to vector–host interaction. In areas of the Peruvian Amazon, a similar trend was also observed, where malaria cases increased because of the development of the roads and ecological changes in correlation to deforestation [48, 49]. In Sabah, East Malaysia, it has also been demonstrated that forest cover and deforestation are risk factors associated with transmission of knowlesi malaria to humans [50, 51]. Thus, the disturbed natural environment has been correlated to changes in non-human primate behavior, increasing their contact with humans [52] eventually exposing humans to more zoonotic malaria. The impact of habitat can also be expressed in elevation associations. Less number of cases was reported near highland areas. Vector densities in highlands were lower than in lowlands. This could be because the temperature in elevated areas was low, which might negatively affect the growth of *Anopheles* mosquitoes, thus leading to lesser transmission in highlands [53, 54].

In malaria elimination, the focus is only on the four species of human malaria [1]. However, with the reduction of human malaria cases, *P. knowlesi* cases are on the increase. Therefore, it could be confusing for the public to comprehend malaria elimination in Malaysia, while zoonotic knowlesi malaria afflicts people. Thus, vectors need to be identified so that suitable control measures can be instituted for successful and complete malaria elimination. Furthermore, due to geographical variations, there will be biological differences between the *Anopheles* species [55] and the environment in which they are adapted. Hence vector distribution maps can be extensively used by the malaria control programs for planning surveillance and control.

*Anopheles* species from the Leucosphyrus Group have been incriminated as vectors of *P. knowlesi* in natural settings [9, 15, 16, 54–59]. However, in Sabah and Sarawak, Malaysian Borneo, *Anopheles donaldi* has been incriminated as vector based on detection of *P. knowlesi* DNA [57, 60]. Furthermore*, P. knowlesi* DNA was also detected in *Anopheles sundaicus* in Andaman and Nicobar islands of India [61]. Therefore, the transmission patterns and vector ecology provide a significant challenge to knowlesi malaria vector control, which is vital for malaria elimination. However, in this study, all *Anopheles* mosquitoes were dissected and examined for sporozoites and oocysts, followed by molecular identification. Furthermore, the finding of two clades of *An. latens*, likely associated with East and West Malaysia points to the need for further detailed taxonomical investigations. Identification of actual vectors involving transmission is of utmost importance.

Additionally, GIS has been used to make spatial analysis useful [62]. GIS produces spatial information on the disease and converts them into a map with helpful information to help explain better the geographic pattern, relationship, and change of the disease. For example, in south Iran, GIS was used to display the spatial distribution of the human malaria case and risk map for that area [63]. Based on the study, the use of tools such as GIS should be strongly suggested as a surveillance tool, especially in areas with high cases. For example, spatial analysis has shown that cases have been highly concentrated in certain districts such as Mersing and Kota Tinggi. However, a random pattern of distribution of cases has been identified in Segamat, Tangkak, Muar, Batu Pahat, and few areas in Mersing and Kota Tinggi. Thus, the current study would allow control and preventive methods to be more targeted, efficient, and cost-effective. Finally, it also shows that for the identification of vectors, the use of molecular techniques is crucial to prevent misidentification. Besides, another simian malaria-like *P. cynomolgi* is reported in Malaysia and Southeast Asia [64].

The limitation of this study is that the macaque distribution data was not available and was not included in the analysis. However, this is an essential factor, and future studies should include macaque distribution when studying zoonotic malaria. Secondly, more sites should be surveyed for vectors and their larval breeding areas to provide concrete data for the management of vectors. Besides, indepth surveys of human population who may be asymptomatic will help to highlight the areas that may be a potential threat to this disease as was demonstrated in a recent study [65]. Thus, in the light of malaria elimination the results of this study further elaborates the need for more extensive data so as to determine the risk of humans to this disease in the State. This will allow more proactive approaches to be carried out to forestall pending outbreaks.

## Conclusions

From this study, it can be inferred that further research work is required to prevent knowlesi malaria cases. It will be hard to plan any effective control strategies unless more information is obtained on the vectors and macaques involved in the transmission. A GIS-based approach that could be effective as an intervention tool to control malaria. Distribution of *An. latens* and *An. introlatus* were found to overlap with the distribution of knowlesi malaria cases, highly indicative of them being the primary vectors for simian malaria in these areas in Johor. This study demonstrates a constant relationship between malaria occurrence and spatial variation to allow vector control efforts aimed at a particular region.

## Supplementary Information

**Additional file 1: Table S1. ***Anopheles* species collection in Johor State, Malaysia from year 2019–2020

## Data Availability

The data that support the findings of this study are available within the manuscript.

## Abbreviations

COI: Cytochrome c oxidase subunit I
ITS2: Internal transcribed spacer 2 region
GIS: Geographic information System
