# Supplementary material for: Spatial distribution of Plasmodium knowlesi cases and their vectors in Johor, Malaysia: in light of human malaria elimination
Source: Malar J. 2021 Oct 29;20:426. doi: 10.1186/s12936-021-03963-0 (PMC8555301; doi:10.1186/s12936-021-03963-0)
Supplement: Supplementary file 1 — Additional file 1: Table S1. Anopheles species collection in Johor State, Malaysia from year 2019–2020 [file 12936_2021_3963_MOESM1_ESM.docx]

**Additional file 1** *Anopheles* species collection in Johor State, Malaysia from year 2019-2020

| Districts | Name of the sites | Species | Total |
| --- | --- | --- | --- |
| Kota Tinggi | Gunung Panti  (Forest) (1°52'18.4"N 103°52'23.2"E) | *An. epiroticus* | 1 |
|  |  | *An. hycarnus* | 2 |
|  |  | *An. introlatus* | 8 |
|  |  | *An. latens* | 17 |
|  |  | *An. letifer* | 193 |
|  |  | *An. maculatus* | 2 |
|  | Kg Sri Delima  (Farm) (1°42'30.2"N 103°49'25.1"E) | *An. barbirostris* | 1 |
|  |  | *An. introlatus* | 1 |
|  |  | *An. letifer* | 2 |
|  | Kg Sri Aman  (Village) (1°52'17.7"N 103°52'25.4"E) | *An. karwari* | 1 |
|  |  | *An. letifer* | 3 |
|  | Kg Orang Asli Sg Layau  (Indigenous settlement) (1°32'36.3"N 104°03'40.8"E) | *An. barbirostris* | 3 |
|  |  | *An. epiroticus* | 11 |
|  |  | *An. maculatus* | 2 |
| Mersing | Kem Microwave  (Forest) (2°17'14.1"N 103°40'27.8"E) | *An. barbirostris* | 1 |
|  |  | *An. introlatus* | 189 |
|  |  | *An. letifer* | 4 |
|  |  | *An. maculatus* | 74 |
|  | Hutan Lenggor  (Forest) (2°17'46.8"N 103°39'58.4"E) | *An. introlatus* | 94 |
|  |  | *An. karwari* | 12 |
|  |  | *An. letifer* | 8 |
|  |  | *An. maculatus* | 20 |
|  | Teluk Buih Pasar Nelayan  (Rural village) (2°30'18.2"N 103°50'16.7"E) | *An. epiroticus* | 5 |
|  | Kongsi Balak Mersing  (Forest fringe) (2°13'28.3"N 103°42'10.8"E) | *An. introlatus* | 73 |
|  |  | *An. maculatus* | 28 |
|  | Kg Orang Asli Berasau  (Plantation) (2°13'01.3"N 103°35'13.0"E) | *An. introlatus* | 1 |
| Kluang | Bkt Kenangan  (Plantation) (1°46'31.6"N 103°21'11.7"E) | *An. barbirostris* | 1 |
|  |  | *An. maculatus* | 46 |
|  | Kg Orang Asli Punjut  (Plantation) (2°14'19.5"N 103°34'46.6"E) | *An. brevipalpis* | 6 |
|  |  | *An. introlatus* | 1 |
|  |  | *An. maculatus* | 6 |
|  | Gunung Lumut  (Forest fringe) (2°04'18.2"N 103°30'49.4"E) | *An. maculatus* | 4 |
